# Elevated ethyl methanesulfonate (EMS) in nelfinavir mesylate (Viracept^®^, Roche): overview

**DOI:** 10.1186/1742-6405-6-18

**Published:** 2009-08-06

**Authors:** Anton Pozniak, Lutz Müller, Miklos Salgo, Judith K Jones, Peter Larson, David Tweats

**Affiliations:** 1 Department of HIV and Genitourinary Medicine, Chelsea and Westminster Hospital, London, UK; 2 Pharmaceuticals Division, F. Hoffmann-La Roche, Basel, Switzerland; 3 The Degge Group, Arlington, VA, USA; 4 Pharmaceuticals Division, F. Hoffmann-La Roche, Nutley, NJ, USA; 5 Medical School, University of Wales, Swansea, UK

## Abstract

Roche's protease inhibitor nelfinavir mesylate (Viracept^®^) produced between March 2007-June 2007 was found to contain elevated levels of ethyl methanesulfonate (EMS), a known mutagen (alkylator) – leading to a global recall of the drug. EMS levels in a daily dose (2,500 mg Viracept/day) were predicted not to exceed a dose of ~2.75 mg/day (~0.055 mg/kg/day based on 50 kg patient). As existing toxicology data on EMS did not permit an adequate patient risk assessment, a comprehensive animal toxicology evaluation of EMS was conducted. General toxicity of EMS was investigated in rats over 28 days. Two studies for DNA damage were performed in mice; chromosomal damage was assessed using a micronucleus assay and gene mutations were detected using the MutaMouse transgenic model. In addition, experiments designed to extrapolate animal exposure to humans were undertaken. A general toxicity study showed that the toxicity of EMS occurred only at doses ≥ 60 mg/kg/day, which is far above that received by patients. Studies for chromosomal damage and mutations in mice demonstrated a clear threshold effect with EMS at 25 mg/kg/day, under chronic dosing conditions. Exposure analysis (C_max_) demonstrated that ~370-fold higher levels of EMS than that ingested by patients, are needed to saturate known, highly conserved, error-free, mammalian DNA repair mechanisms for alkylation. In summary, animal studies suggested that patients who took nelfinavir mesylate with elevated levels of EMS are at no increased risk for carcinogenicity or teratogenicity over their background risk, since mutations are prerequisites for such downstream events. These findings are potentially relevant to >40 marketed drugs that are mesylate salts.

## Findings

Nelfinavir mesylate (Viracept^®^) is a protease inhibitor for the treatment of HIV-infected patients produced by Roche outside the US, Canada and Japan (in these regions it is manufactured by Pfizer and marketed by Pfizer or Japan Tobacco). It was first introduced by Roche in 1998 following FDA approval in 1997. Although newer protease inhibitors have become available for the treatment of HIV, nelfinavir is seen as a useful medicine for: HIV-infected patients who are intolerant to ritonavir, since it does not require ritonavir PK enhancement (boosting), HIV-infected pregnant women, and HIV patients in resource-limited settings since the formulation is heat-stable and does not require refrigeration.

In mid-May 2007, Roche received reports from France and Spain of a strange odour associated with bottles of nelfinavir, including one patient reporting nausea and vomiting. Investigations revealed that there were elevated levels of ethyl methanesulfonate (EMS) in nelfinavir batches produced between March 2007 and May 2007. Further investigation revealed this to be due to manufacturing issues, more specifically failure to dry the hold tank following ethanol cleaning. As EMS is a known alkylating agent that acts on DNA to produce mutagenic and carcinogenic effects in animals, this led to a global recall of the drug on June 6, 2007 facilitated via an extensive communication programme managed by Roche. Subsequent retrospective testing of all nelfinavir batches produced since 1998 showed negligible levels (<1 ppm) in most batches but a few incidences in May 2004 and June 2005 of the presence of approximately 100 ppm of EMS prior to March 2007 [1].

Based on the highest amount of EMS found in nelfinavir tables (920 ppm), the EMS levels in a daily dose of nelfinavir (2,500 mg nelfinavir/day [10 tablets]) were shown to result in a daily dose of EMS no higher than ~2.75 mg/day (~0.055 mg/kg/day based on the conservative assumption of a 50 kg patient). As it was crucial to understand the potential risks to patients exposed to this level of EMS over this period, Roche (with agreement from the health authorities) designed both a comprehensive pre-clinical toxicology programme and safety follow-up registries of exposed patients.

### Pre-clinical program

EMS is formed from methanesulfonic acid and ethanol; it is not thought to occur in nature and has no commercial uses. EMS is, however, a known trace impurity in pharmaceuticals formulated as mesylate salts and for this reason it is considered an impurity rather than a contaminant. EMS is capable of inducing gene mutations and chromosomal aberrations via alkylation, mainly at the O^6 ^position of the guanine base [2]. Alkylation at this site of the DNA is repaired (error-free repair) by a specific cellular suicide "enzyme" called O^6^-methyl guanine methyltransferase (MGMT) [2]. Human risk assessments for direct alkylating agents such as EMS are normally based on a linear dose response, i.e. a 'safe dose level' (threshold) can not be calculated. However, recently published *in vitro *data by Doak et al., indicated a threshold response at low doses of EMS [3].

The pre-clinical programme focused on three key areas: the general toxicity of EMS, the genotoxicity of EMS and the subsequent analysis to extrapolate the findings to humans.

### General toxicity study

A general toxicity study was undertaken in rats and was designed to identify any potential organ toxicities and clinical chemistry/haematology changes. EMS was administered daily by oral gavage to rats of both sexes at dose levels of 20, 60 and 180 mg/kg/day for a period of 28 days [4]. No adverse effects were observed in rats at a dose of 20 mg/kg/day (compared to the highest potential patient exposure of EMS of 0.055 mg/kg/day). At doses of 60 mg/kg/day or above, changes associated with toxicity such as decreased cell counts, and decreased organ weight/size were seen in bone marrow, blood cells, testes and lymphatic tissues (as would be expected for an alkylating agent) [4]. Importantly, no necrotic or pre-cancerous lesions, nor major changes in laboratory values, were observed even at the highest dose, over the 28 days.

### Genotoxicity studies

Two separate assays were used to assess the genotoxicity of EMS. Firstly, a micronucleus test (MNT) in bone marrow cells of mice was conducted to investigate the dose-response of chromosome damage induction at low doses of EMS [5]. Secondly, a large MutaMouse™ study was undertaken to investigate the dose-response of gene mutation induction in the transgenic lacZ reporter gene at low doses of EMS [5].

**Figure 1:**
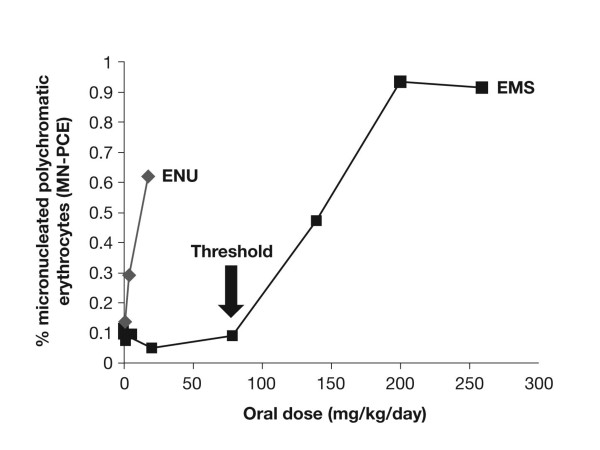
**EMS and ENU: induction of micronuclei in mouse bone marrow after 7 days of treatment**.

The MNT assay was performed as previously described [5]. The results are shown in Figure 1. The positive control, ethyl nitrosourea (ENU), a genotoxic agent that induces a similar spectrum of DNA adducts but in distinctively different proportions than EMS [6], demonstrated a clear linear response with regards to micronucleus induction. In contrast, EMS showed a threshold dose-response with no micronuclei induction at EMS doses up to, and including, 80 mg/kg/day.

### MutaMouse assay

**Figure 2:**
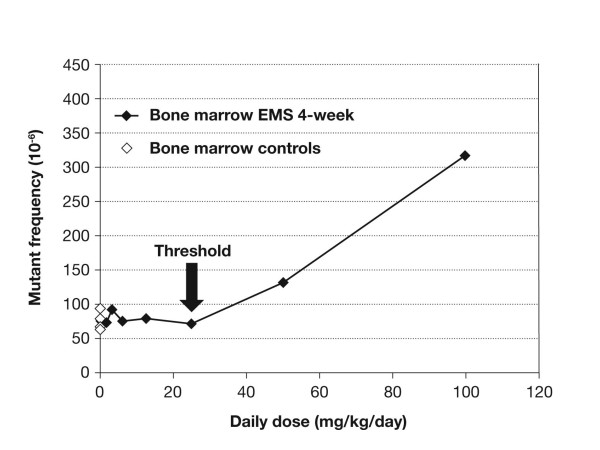
**Induction of lacZ mutations in bone marrow as a function of dose**.

**Figure 3:**
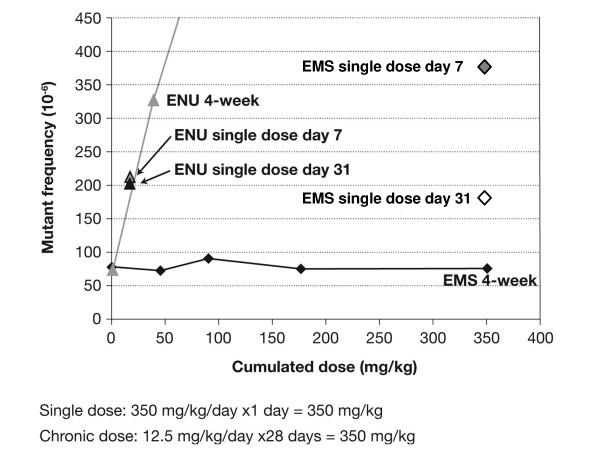
**Induction of LacZ mutations in bone marrow after single dose and 4-week chronic dosing with EMS and ENU (effects plotted against the cumulative dose)**.

The MutaMouse assay – an assay that uses transgenic mice to evaluate the induction of specific gene mutations – was performed as previously described [5]. Similar to that observed with the MNT, the MutaMouse study demonstrated a clear threshold effect for mutations with EMS at 25 mg/kg/day in both bone marrow (Figure 2) and gastrointestinal tract, and 50 mg/kg/day in liver tissue (data not shown), under chronic dosing conditions. These experiments also indicated that the genotoxic effect of ENU is independent of dose fractionation – i.e., when the dose is given at one time or over 30 days (Figure 3). In contrast, for EMS the single dose treatment (350 mg/kg) clearly induced mutations whereas the 4-week, chronic dosing with 12.5 mg/kg/day for 30 days (total dose 350 mg) showed no mutation induction – suggesting that any mutations that are induced are repaired via the MGMT repair mechanism. The results of the genotoxicity studies described here were probed with statistical methods, which yielded clear evidence for a hockey-stick dose-response relationship, i.e. a dose-response with a threshold below which exposure occurs but no genotoxic (and hence no teratogenic or carcinogenic) effect versus the spontaneous background is seen. The assumption of linearity could be reliably refuted for all parts of the studies [7].

The last phase of the pre-clinical program was to extrapolate these findings to humans (in the absence of obtaining human measurements) to facilitate a retrospective exposure determination in patients having taken nelfinavir containing elevated levels of EMS. The first step was to generate a reliable EMS PK model by administering EMS both orally and intravenously to mice and rats. The results showed that EMS exhibits a high oral bioavailability (~100%), high clearance (~0.6 mL/min/kg) and limited volume of distribution corresponding to total body water (~0.5 L/kg). The model was then validated using actual data (oral and intravenous administration) from mice (data not shown).

The availability of this comprehensive PK model allowed for the estimation of the EMS PK profile in humans in which a conservative scenario was assumed with 100% bioavailability and a very low clearance. Using this model the following parameters were calculated: C_max _= 0.85 μM, AUC = 13 μM*h and half-life = 11 hr, a half-life similar to that of EMS in buffer alone [8,9].

**Figure 4:**
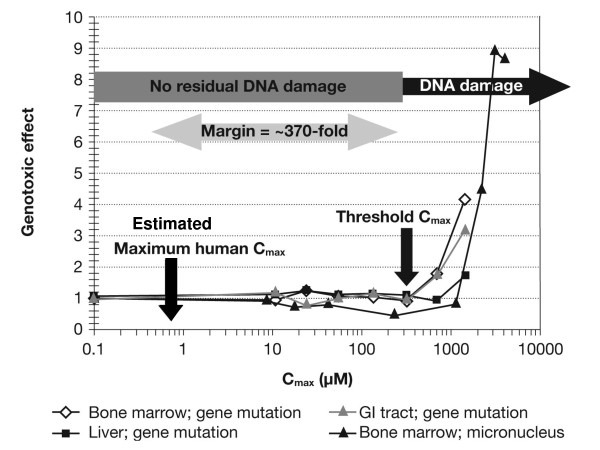
**Risk assessment for Viracept patients calculated using C_max _(measure of peak exposure)**.

Comparing the retrospectively calculated exposure (C_max_) of humans (0.85 μM) to free EMS (in nelfinavir) with the exposure of EMS at the threshold based on the animal experiments (315 μM) showed that ~370-fold higher levels of EMS, than that ingested by patients (based on the conservative estimation of an EMS dose in nelfinavir of 0.055 mg/kg/day), are needed to saturate known, highly-conserved, error-free, mammalian DNA repair mechanisms for alkylation (Figure 4) [10].

For an error-free repair mechanism of DNA damage, C_max _is most likely the relevant determinant for safety margin assessment at doses below the threshold. However, the safety margin was also calculated based on the more conservative exposure measure of AUC and this showed that ~30-fold higher levels of EMS, than that ingested by patients were required to saturate the DNA repair mechanisms (data not shown) [10].

## Conclusion

Extensive animal studies, which were deemed relevant and predictive for the human situation by the European Medicines Agency (EMEA), indicate that EMS exhibits a threshold response for chromosome damage and mutations when administered at doses below 25 mg/kg/day. This threshold dose is far above that estimated as a worse case to have been received by nelfinavir patients (0.055 mg/kg/day). Following extrapolation of these animal data to humans, it was demonstrated that even at an exposure level of 30 to 370-fold higher than that ingested by nelfinavir patients, the damage incurred by EMS on DNA can still effectively be dealt with by DNA repair mechanisms. Since chromosome damage and mutations are the underlying molecular events for teratogenicity and carcinogenicity of alkylating agents like EMS, experts and the EMEA agreed that the studies above could be used to assess these risks for potentially affected nelfinavir patients, with adequate reassurance on margins of safety.

In light of these extensive data – both independent experts in toxicology, epidemiology and HIV clinical management, together with the EMEA's Committee for Medicinal Products for Human Use (CHMP), recommended that retrospective patient registries were not required as there was no indication that patients had been exposed to levels of EMS that would result in permanent DNA damage. In the context of pharmacovigilance for marketed drugs, this was the first case in which a post-marketing safety event was completely and thoroughly addressed with animal toxicological investigations obviating the need for patient registries [11].

## Competing interests

AP has acted as a consultant for Roche Pharmaceuticals Ltd.

LM, MS and PL are full time employees of Roche Pharmaceuticals.

JKJ serves as a consultant to and/or conducts epidemiology studies for several pharmaceutical companies, including Roche Pharmaceuticals, Bayer, Abbott Laboratories, Genentech, Allergan, sanofi-aventis, Cephalon, Nycomed, Lundbeck and Astellas Pharma.

DT has acted as a consultant for Roche Pharmaceuticals Ltd.

## Authors' contributions

LM, MS, JKJ and PL conceived of the study, and participated in its design and coordination and helped draft the manuscript. AP reviewed the study findings from a clinical standpoint and helped draft the manuscript. DT reviewed the study findings from a toxicological standpoint and helped draft the manuscript. All authors read and approved the final manuscript.
